# Development and validation of a nomogram to predict the risk of peripheral artery disease in patients with type 2 diabetes mellitus

**DOI:** 10.3389/fendo.2022.1059753

**Published:** 2022-12-12

**Authors:** Jiemei Liang, Jiazhao Song, Tiehui Sun, Lanning Zhang, Shan Xu

**Affiliations:** ^1^ Hebei North University, Zhangjiakou, Hebei, China; ^2^ Air Force Medical Center, PLA, Beijing, China

**Keywords:** type 2 diabetes mellitus (T2DM), peripheral artery disease (PAD), nomogram, risk factor, predictive model

## Abstract

**Objective:**

To develop and validate a nomogram for predicting the risk of peripheral artery disease (PAD) in patients with type 2 diabetes mellitus (T2DM) and assess its clinical application value.

**Methods:**

Clinical data were retrospectively collected from 474 patients with T2DM at the Air Force Medical Center between January 2019 and April 2022. The patients were divided into training and validation sets using the random number table method in a ratio of 7:3. Multivariate logistic regression analysis was performed to identify the independent risk factors for PAD in patients with T2DM. A nomogram prediction model was developed based on the independent risk factors. The predictive efficacy of the prediction model was evaluated using the consistency index (C-index), area under the curve (AUC), receiver operating characteristic (ROC) curve, Hosmer-Lemeshow (HL) test, and calibration curve analysis. Additionally, decision curve analysis (DCA) was performed to evaluate the prediction model’s performance during clinical application.

**Results:**

Age, disease duration, blood urea nitrogen (BUN), and hemoglobin (*P*<0.05) were observed as independent risk factors for PAD in patients with T2DM. The C-index and the AUC were 0.765 (95% CI: 0.711-0.819) and 0.716 (95% CI: 0.619-0.813) for the training and validation sets, respectively, indicating that the model had good discriminatory power. The calibration curves showed good agreement between the predicted and actual probabilities for both the training and validation sets. In addition, the *P*-values of the HL test for the training and validation sets were 0.205 and 0.414, respectively, indicating that the model was well-calibrated. Finally, the DCA curve indicated that the model had good clinical utility.

**Conclusion:**

A simple nomogram based on three independent factors–duration of diabetes, BUN, and hemoglobin levels–may help clinicians predict the risk of developing PAD in patients with T2DM.

## Introduction

Diabetes mellitus (DM) is a group of metabolic diseases characterized by hyperglycemia, the prevalence of which is increasing worldwide. In 2019, the ninth edition of the Diabetes Atlas of the International Diabetes Federation (IDF) indicated that there were approximately 463 million patients with diabetes worldwide, which is expected to increase to 700 million by 2045 (1).

Type 2 DM (T2DM) is a non-insulin-dependent DM that accounts for 90–95% of all DM cases. The main feature of T2DM is a progressive decrease in insulin secretion by beta cells (2). Peripheral artery disease (PAD) is a general term for all vascular diseases that result in functional and structural abnormalities of the aorta, its branches, and lower extremity arteries, secondary to atherosclerosis- and thromboembolism-related pathophysiology (3). PAD is characterized by occlusion of the lower-extremity arteries (4), most commonly the femoral, popliteal, tibial, and peroneal arteries, leading to a higher risk of lower-extremity amputation (5).

PAD in patients with T2DM presents a wide range of clinical features and consequences and is known as one of the major macrovascular complications of T2DM (6). According to reports, T2DM significantly increases the incidence of PAD, the risk of ischemic events and amputations (7), and disease progression and severity (8). The resulting severe disability and cardiovascular risk in patients with diabetes lead to a sharp decline in their quality of life and a rapid increase in socioeconomic burden (9).

However, in individuals with diabetes, PAD awareness is still suboptimal; this is partly related to the atypical clinical presentation of PAD in some cases. Therefore, regular and appropriate PAD screening is recommended for patients with T2DM. Currently, PAD diagnosis mainly relies on imaging examinations, such as B-ultrasound and magnetic resonance imaging (MRI), and the related risk factors have received little attention. Hence, further research is required to improve our understanding of the PAD risk factors.

Nomograms are developed using individual risk factors identified by multiple logistic regression analyses. They can graphically represent the numerical relationship between specific diseases and their risk factors and intuitively predict the incidence of adverse events through a scoring system without any complicated calculation formulas (10). In addition, nomograms can provide accurate and personalized risk predictions for each individual, allowing clinicians to effectively screen high-risk patients and provide timely interventions. Therefore, this study aimed to develop a nomogram prediction model for the risk of peripheral vascular disease in patients with T2DM to screen and identify high-risk patients early and provide a reliable reference for early clinical intervention.

## Material and methods

### Patients selection

**Figure 1:**
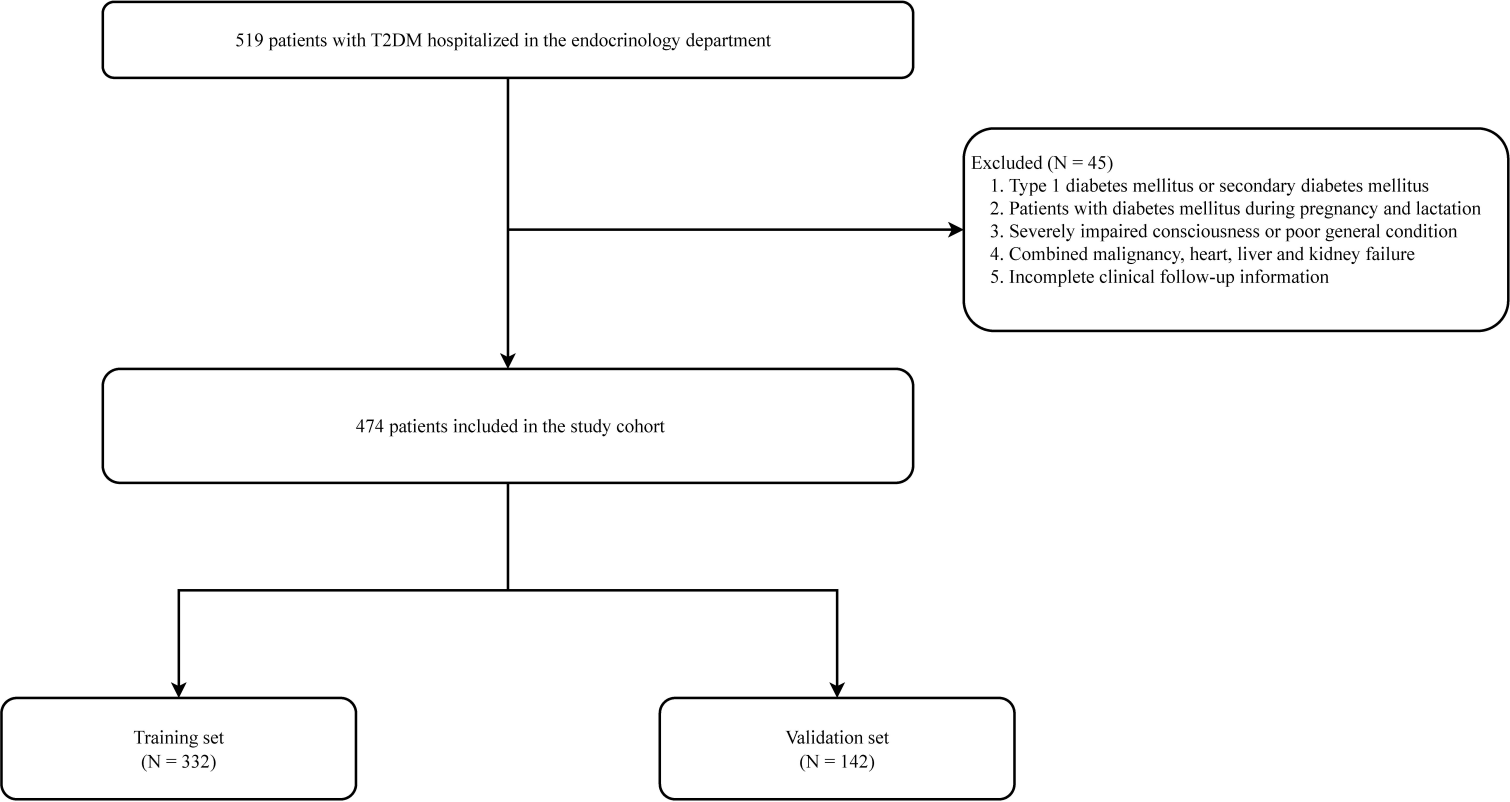
Flow chart for peripheral artery disease in patients with type 2 diabetes mellitus patients.

Information was collected from patients with T2DM in the Air Force Medical Center’s hospital database from January 2019 to April 2022 (**Figure 1**). The diagnostic criteria for T2DM were based on the diagnostic criteria and classification of the American Diabetes Association (ADA) (11). PAD diagnosis was determined according to the current guidelines in conjunction with ancillary tests. The tests included ankle-brachial index (ABI), Doppler ultrasound of extremity vessels, computed tomography angiography, vascular MRI, or arteriography. The inclusion criteria were as follows (1): patients with confirmed T2DM and (2) patients aged >18 years old. The exclusion criteria were as follows: patients with (1) type 1 diabetes (T1DM) or secondary diabetes; (2) diabetes during pregnancy and lactation; (3) severely impaired consciousness or poor general condition; (4) combined malignancy and cardiac, hepatic, and renal failure; and (5) incomplete clinical follow-up information.

### Observation variables

Clinical variables observed included: age, sex, height, body weight, body mass index (BMI), diastolic blood pressure (DBP), systolic blood pressure (SBP), duration of T2DM, history of smoking, history of drinking, coronary heart disease (CHD), high blood pressure (HBP), hyperlipidemia, glycated hemoglobin (HbA1c), fasting blood glucose (FBG), blood urea nitrogen (BUN), serum creatinine (Scr), blood uric acid (BUA), total cholesterol (TC), triglycerides (TG), high-density lipoprotein cholesterol (HDL-C), low-density lipoprotein cholesterol (LDL-C), alanine aminotransferase (ALT), aspartate aminotransferase (AST), albumin (ALB), white blood cell count (WBC), hemoglobin (Hb), and red blood cell-specific volume (HCT). Data were collected from blood samples taken after 8 h of fasting on the day following the patient’s admission to the hospital. Furthermore, the authors checked the above data individually to determine the completeness of the data.

### Building and validating the nomogram

Data from hospitalized patients with T2DM were randomized into training and validation sets at a 7:3 ratio. A nomogram was created using independent risk factors to predict the probability of PAD in patients with T2DM. The consistency index (C-index), area under the curve (AUC), receiver operating characteristic (ROC) curve, and calibration curve were used to evaluate the predictive reliability and predictive ability of the nomogram, respectively. The Hosmer-Lemeshow (HL) test was used to verify that the model was well-calibrated. The nomogram was validated using the bootstrap method with 1000 resamples and a validation set to further evaluate the model’s applicability. In addition, the clinical usability of the nomogram was estimated using decision curve analysis (DCA).

### Statistical analysis

Categorical variables were expressed as numbers and percentages (%), and continuous variables were expressed as mean (SD). Categorical variables were analyzed using the Chi-square test, while continuous variables were compared using the student’s t-test or Mann-Whitney U test. Multivariate logistic regression was used to identify the independent risk factors for PVD in patients with T2DM. All analyses were performed using R v.4.4.1, and statistical software (http://www.R-project.org, The R Foundation, Vienna, Austria) was used to comprehensively analyze the collected data. A two-tailed *P-*value <0.05 was considered statistically significant.

## Results

### Basic characteristics of the population

This study ultimately included 474 patients, with the training and validation sets comprising 332 and 142 patients, respectively (**Figure 1**; **Table 1**). In the training set, 212 patients developed peripheral vascular lesions with a prevalence of 63.9%. For all variables included in the analysis, there was no statistically significant difference between the training and validation sets (*P*<0.05), indicating comparability between the two groups.

**Table 1:** Baseline characteristics of patients in the training set and validation set.

Variables	Total (n = 474)	Training set (n = 332)	Validation set (n = 142)	*P* value
Age (years)	64.0 ± 10.6	63.9 ± 10.9	64.4 ± 9.9	0.643
Sex, n (%)				0.469
Male	302 (63.7)	215 (64.8)	87 (61.3)	
Female	172 (36.3)	117 (35.2)	55 (38.7)	
Height (cm)	168.2 ± 8.1	168.2 ± 8.2	168.1 ± 7.6	0.859
Body weight (kg)	70.1 ± 11.9	70.6 ± 12.3	68.9 ± 11.0	0.174
BMI, n (%)	24.7 ± 3.7	24.9 ± 4.0	24.3 ± 2.9	0.111
DBP (mmol/L)	78.0 ± 11.4	78.4 ± 10.6	77.2 ± 13.1	0.313
SBP (mmol/L)	133.0 ± 17.8	133.6 ± 17.5	131.6 ± 18.5	0.264
Duration of T2MD (months)	15.9 ± 9.2	15.6 ± 9.4	16.7 ± 8.6	0.225
Smoking, n (%)				0.583
No	286 (60.3)	203 (61.1)	83 (58.5)	
Yes	188 (39.7)	129 (38.9)	59 (41.5)	
Drinking, n (%)				0.452
No	309 (65.2)	220 (66.3)	89 (62.7)	
Yes	165 (34.8)	112 (33.7)	53 (37.3)	
CHD, n (%)				0.557
No	336 (70.9)	238 (71.7)	98 (69)	
Yes	138 (29.1)	94 (28.3)	44 (31)	
HBP, n (%)				0.127
No	140 (29.5)	105 (31.6)	35 (24.6)	
Yes	334 (70.5)	227 (68.4)	107 (75.4)	
Hyperlipidemia, n (%)				0.733
No	236 (49.8)	167 (50.3)	69 (48.6)	
Yes	238 (50.2)	165 (49.7)	73 (51.4)	
HbA1c (%)	8.8 ± 2.0	8.8 ± 2.0	8.7 ± 1.8	0.789
FBG (mmol/L)	8.6 ± 3.2	8.6 ± 3.3	8.7 ± 2.8	0.763
BUN (mmol/L)	7.0 ± 2.9	7.1 ± 3.0	6.8 ± 2.6	0.309
Scr (μmol/L)	84.4 ± 78.2	85.4 ± 83.1	82.3 ± 65.3	0.693
BUA (μmol/L)	321.9 ± 89.8	320.1 ± 89.3	326.0 ± 90.9	0.512
TC (mmol/L)	4.0 ± 1.1	4.0 ± 1.0	3.9 ± 1.1	0.403
TG (mmol/L)	1.7 ± 1.0	1.7 ± 1.0	1.6 ± 0.9	0.647
HDL-C (mmol/L)	1.0 ± 0.3	1.0 ± 0.3	1.0 ± 0.3	0.932
LDL-C (mmol/L)	2.2 ± 0.8	2.3 ± 0.8	2.2 ± 0.8	0.608
ALT (U/L)	18.5 ± 18.7	17.8 ± 11.6	20.2 ± 29.2	0.184
AST (U/L)	18.1 ± 8.2	18.3 ± 7.8	17.9 ± 9.0	0.626
ALB (g/L)	40.0 ± 5.1	39.8 ± 5.1	40.3 ± 5.0	0.426
WBC (×10^9/L)	6.8 ± 2.1	6.7 ± 2.0	6.9 ± 2.2	0.350
Hb	122.0 ± 22.0	122.4 ± 21.3	121.0 ± 23.6	0.519
HCT (%)	0.4 (0.3, 0.4)	0.4 (0.3, 0.4)	0.4 (0.3, 0.4)	0.940

BMI, body mass index; DBP, diastolic blood pressure; SBP, systolic blood pressure; T2DM, type 2 diabetes mellitus; CHD, coronary heart disease; HBP, high blood pressure; HbA1c, glycated hemoglobin; FBG, fasting blood glucose; BUN, blood urea nitrogen; Scr, serum creatinine; BUA, blood uric acid; TC, total cholesterol; TG, triglycerides; HDL-C, high-density lipoprotein cholesterol; LDL-C, low-density lipoprotein cholesterol; ALT, alanine aminotransferase; AST, aspartate aminotransferase; ALB, albumin; WBC, white blood cell count; Hb, hemoglobin; HCT, red blood cell specific volume.

### Risk factors screening

Univariate logistic regression analysis showed that age, BMI, DBP, duration of T2DM, CHD, HBP, BUN, Scr, TC, TG, HDL-C, LDL-C, AST, ALB, and Hb were significantly different risk factors in the training set (*P*<0.05, **Table 2**). These risk factors were included in the multivariate logistic regression analysis, which showed that the independent risk factors for a diabetic foot in patients with T2DM were disease duration, BUN, and Hb (*P*<0.05, **Table 2**).

**Table 2:** Univariate and multivariate logistic regression analyses for patients with T2DM.

Variable	OR 95%CI	*P* value	OR 95%CI	*P* value
Age (years)	1.06 (1.04~1.08)	<0.001	1.01 (0.99~1.04)	0.396
Sex, n (%)				
Male				
Female	0.76 (0.51~1.12)	0.161		
Height (cm)	1.01 (0.99~1.04)	0.268		
Body weight (kg)	0.99 (0.98~1.01)	0.297		
BMI, n (%)	0.94 (0.89~0.99)	0.028	0.97 (0.91~1.04)	0.437
DBP (mmol/L)	1.01 (1~1.02)	0.143		
SBP (mmol/L)	0.97 (0.95~0.98)	<0.001	0.99 (0.97~1.02)	0.600
Duration of T2MD (months)	1.08 (1.06~1.11)	<0.001	1.05 (1.02~1.07)	0.002
Smoking, n (%)				
No				
Yes	1.1 (0.75~1.63)	0.625		
Drinking, n (%)				
No				
Yes	1.17 (0.78~1.75)	0.451		
CHD, n (%)				
No				
Yes	2.73 (1.7~4.39)	<0.001	1.56 (0.91~2.68)	0.106
HBP, n (%)				
No				
Yes	1.75 (1.17~2.64)	0.007	1.15 (0.7~1.89)	0.586
Hyperlipidemia, n (%)				
No				
Yes	0.69 (0.47~1.02)	0.061		
HbA1c (%)	0.98 (0.89~1.08)	0.690		
FBG (mmol/L)	1.05 (0.98~1.11)	0.159		
BUN (mmol/L)	1.25 (1.13~1.37)	<0.001	1.16 (1.02~1.31)	0.020
Scr (μmol/L)	1.01 (1~1.02)	0.001	1 (0.99~1.01)	0.718
BUA (μmol/L)	1 (1~1)	0.161		
TC (mmol/L)	0.75 (0.63~0.9)	0.002	1.19 (0.58~2.47)	0.634
TG (mmol/L)	0.82 (0.67~0.99)	0.036	0.79 (0.54~1.18)	0.250
HDL-C (mmol/L)	0.36 (0.18~0.71)	0.003	0.31 (0.1~1)	0.050
LDL-C (mmol/L)	0.72 (0.57~0.92)	0.008	0.81 (0.36~1.81)	0.611
ALT (U/L)	0.99 (0.97~1)	0.057		
AST (U/L)	0.96 (0.94~0.98)	0.001	0.97 (0.95~1)	0.050
ALB (g/L)	0.93 (0.89~0.97)	<0.001	1.02 (0.97~1.08)	0.447
WBC (×10^9/L)	1.06 (0.97~1.16)	0.221		
Hb	0.97 (0.96~0.98)	<0.001	0.98 (0.97~1)	0.019
HCT (%)	1.03 (0.93~1.14)	0.537		

BMI, body mass index; DBP, diastolic blood pressure; SBP, systolic blood pressure; T2DM, type 2 diabetes mellitus; CHD, coronary heart disease; HBP, high blood pressure; HbA1c, glycated hemoglobin; FBG, fasting blood glucose; BUN, blood urea nitrogen; Scr, serum creatinine; BUA, blood uric acid; TC, total cholesterol; TG, triglycerides; HDL-C, high-density lipoprotein cholesterol; LDL-C, low-density lipoprotein cholesterol; ALT, alanine aminotransferase; AST, aspartate aminotransferase; ALB, albumin; WBC, white blood cell count; Hb, hemoglobin; HCT, red blood cell specific volume.

### Creation and validation of the nomogram

**Figure 2:**
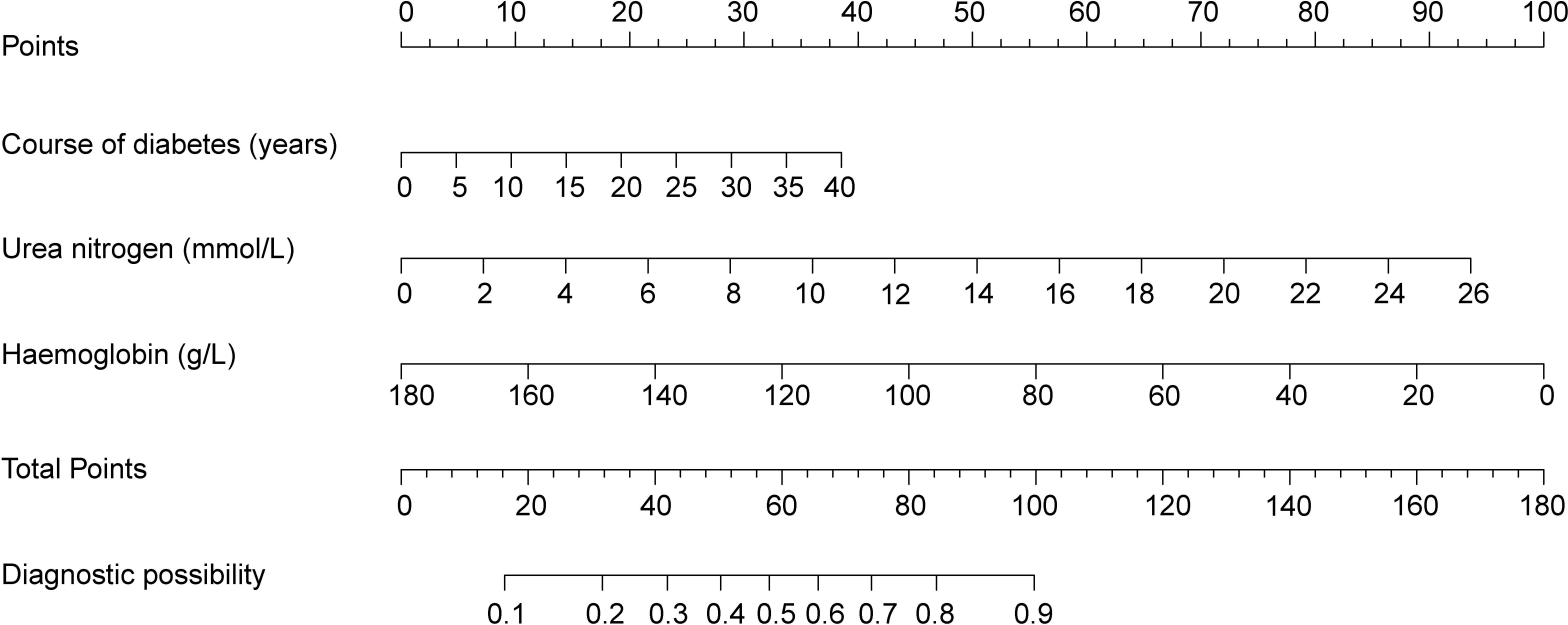
Nomogram predicting peripheral artery disease possibility of type 2 diabetes mellitus patients.

**Figure 3:**
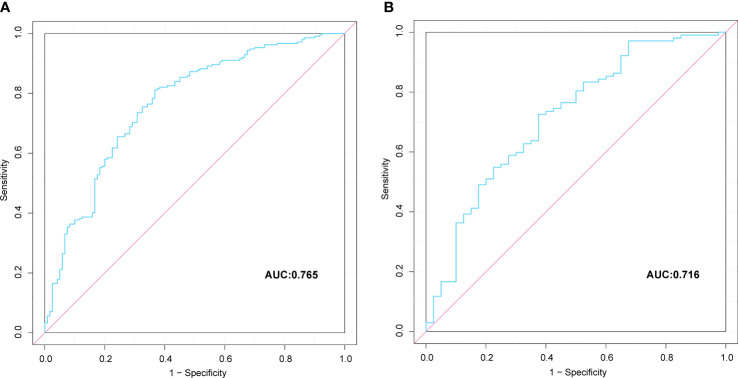
Comparison of the ROC curves of the nomogram for peripheral artery disease possibility prediction in the training set **(A)**, and in the verification set **(B)**.

A nomogram prediction model based on the independent risk factors (**Figure 2**) was developed to predict the risk of concomitant PAD in patients with T2DM. ROC curves of the training and validation sets were drawn. The AUC were 0.765 (95% CI: 0.711–0.819), and 0.716 (95% CI: 0.619–0.813) for the training and validation sets, respectively (**Figures 3A, B**). The C-index of the nomogram in the training and validation sets were >0.70, indicating that the model had a good discriminative ability.

**Figure 4:**
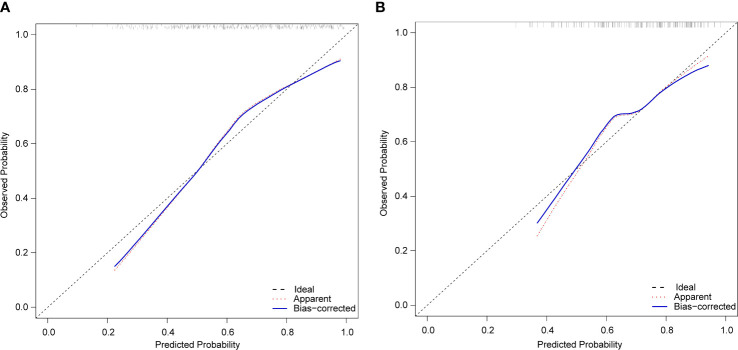
Calibration plots of the nomogram for peripheral artery disease prediction of the training set **(A)** and verification set **(B)**.

The calibration curves of the nomogram in the training and validation sets showed a favorable consistency between the predicted and actual probabilities (**Figure 4**). In addition, the results of the HL test of the nomogram in the training and validation sets were *χ* ^2^ = 10.940 (*P*=0.205) and *χ* ^2^ = 8.197 (*P*=0.414), respectively. The *P*-value of the HL test was insignificant for the model in both the training and validation sets; this indicated that the nomogram fit well.

**Figure 5:**
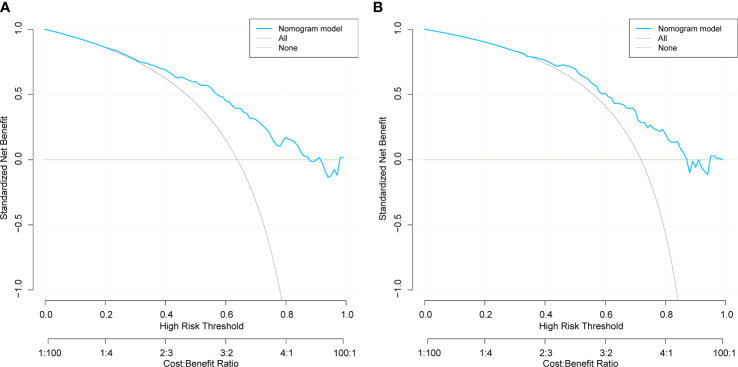
Decision curve analysis of training set **(A)** and validation set **(B)** for the risk of peripheral artery disease in patients with type 2 diabetes mellitus..

The DCA results for the training and validation sets are shown in **Figure 5**. The net benefit for patients was higher than those of the other two curves when the threshold probabilities were 40– 80% and 50– 80%, respectively. The horizontal line indicates that none of the patients developed peripheral vascular lesions, all were untreated, and no intervention benefit was generated. The diagonal line indicates that all patients developed peripheral vascular lesions and the benefit after treatment. Within these ranges, the nomogram prediction model had good clinical utility.

### Clinical application of the nomogram

**Figure 6:**
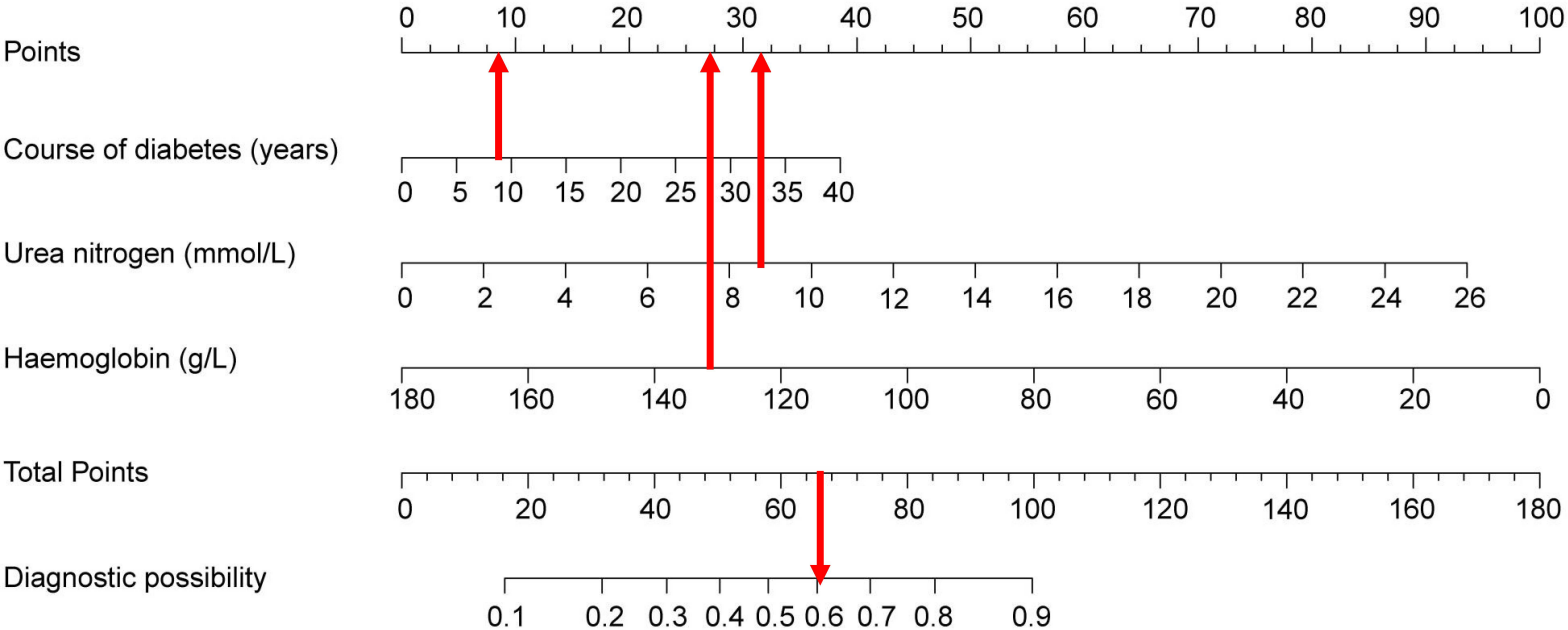
Visualization of nomogram to predict the risk of concomitant peripheral artery disease in patients with type 2 diabetes mellitus patients.

As an example, the basic information of a male patient with a 9-year history of T2DM had a test measurement of urea nitrogen of 8.9 mmol/L and a Hb value of 139 g/L. Combined with the legend, the total score was approximately 66. The corresponding risk of peripheral vascular disease was approximately 60–61% (**Figure 6**). Hence, it is recommended that this patient be examined for peripheral vascularity to detect the onset of lesions and be administered aggressive intervention to prevent PAD.

## Discussion

This retrospective study was based on predicting the risk of PAD in patients with T2DM in the Beijing area. The duration of T2DM and Hb and BUN levels were independent risk factors for PAD. The duration of T2DM and BUN were positively correlated with PAD, and Hb was negatively correlated with PAD. The original data were randomly divided into development (n=332) and validation (n=142) groups. The verification results show that both have good risk-prediction abilities. The calibration plot showed that the nomogram was accurate in predicting T2DM risk, and the DCA also demonstrated the clinical utility of the nomogram.

As a common diabetes-related complication, PAD affects about 10–20% of people with diabetes (12), and in developed countries, about 50%. PAD increases with age in patients with diabetes, and arterial occlusion often occurs in distal limbs that cannot be revascularized (13). It is estimated that PAD is present in up to 50% of diabetic foot ulcers and is an independent risk factor for its development (14, 15). Furthermore, diabetes is associated with a significant risk of diabetes-related foot disease (DFD), with a lifetime incidence of foot ulcers as high as 34%. Moreover, diabetes is the leading cause of amputation (16). When associated with diabetes, PAD has a more severe disease process, a higher likelihood of distal ischemic ulceration and extensive tissue loss, and an increased risk of amputation (17). Early PAD diagnosis and treatment are critical in patients with DM because of the increased risk of nonunion, infection, and amputation of the lower extremities, increased incidence of cardiovascular complications such as myocardial infarction and stroke, and a 5-year mortality rate of >50% (18–20).

In this study, PAD prevalence in patients with diabetes was 63.9%, higher than that in past reports and >10% higher than that in developed countries. We speculate that the high prevalence of PAD in our study population may be due to several factors. First, although our research unit was a specialized hospital, primary medical institutions referred most admitted patients, and their condition was relatively serious. Moreover, the hospital is well-equipped for examinations and can perform more comprehensive examinations. Second, most of this study’s patients were from northern China, which may have been related to their diet and living habits. In addition, PAD prevalence varies with the diagnostic method used (the presence of intermittent claudication, palpation of vessels, or ABI) (21), which is a factor that cannot be ignored. However, foot examination has a limited role in diagnosing PAD. Moreover, many patients with diabetes and PAD have sensitive neuropathy and markedly reduced or completely blocked perception of ischemic pain (22). Hence, many potential patients with PAD have not received attention. Globally, total healthcare expenditures associated with PAD and DFD are very expensive, placing enormous pressure on patient families and national healthcare systems. Therefore, given the adverse consequences of PAD in T2DM patients, it is important to predict its risk. This study aimed to construct a risk-scoring model that could be used in clinical settings to predict the risk of PAD in patients with T2DM. Furthermore, the developed nomogram model can help clinicians identify the risk factors for PAD early, develop appropriate treatment plans, and take targeted measures to prevent morbidity and mortality.

DM is associated with many complications, of which chronic vascular complications have the most complex and important consequences. Furthermore, about 50–80% of people with diabetes die from cardiovascular disease (including coronary artery disease, stroke, peripheral vascular disease, and other vascular diseases). This has a major impact on health care, making cardiovascular diseases a leading cause of morbidity and mortality in people with diabetes (23). Previous studies have also confirmed that age, diabetes duration, and peripheral neuropathy are associated with an increased risk of PAD in patients with pre-existing DM (24–26). The present study showed that the duration of diabetes could be used to predict the risk of PAD, which is consistent with the studies mentioned above.

T2DM causes various macrovascular complications, including hyperglycemia and insulin resistance, *via* different pathogenic pathways (27). Hyperglycemia may regulate vascular inflammation, cytokines, macrophage activation, and growth factor gene expression. Furthermore, it may interfere with normal angiogenesis, collateral artery formation, and muscle repair and affect local regenerative function (28, 29). Insulin resistance causes persistent hyperglycemia and accelerates disease progression. With the prolongation of the course of diabetes, a series of chronic complications occur, such as diabetic peripheral neuropathy (DPN), nephropathy, and retinopathy. DPN and PAD are closely related among these complications, and DPN may usually precede PAD. However, arterial stenosis aggravates DPN (30). Thus, the relationship appears bidirectional. The strong correlation between them suggests that more attention should be paid to patients with PAD in clinical settings, especially those with a long history of diabetes. Furthermore, careful inquiries should be made about their neurological symptoms, and detailed examinations should be conducted to achieve early detection and treatment.

BUN is the main end-product of human protein metabolism and is one of the main indicators of renal function. Studies on BUN in patients with coronary heart disease have shown a strong association between long-term mortality and acute ST-elevation myocardial infarction (STEMI). Researchers believe that BUN is an alternative marker for the kidneys’ response to systemic hemodynamic changes associated with the pathophysiological mechanisms of heart failure (31). Extensive research in this field has established BUN as an independent predictor of mortality in patients with heart failure (32). An elevated BUN level is thought to be a predictor of short-term (within 1 year) and long-term (5.3 years) survival in these patients, even better than creatinine and other parameters that reflect renal insufficiency (33, 34). A cross-sectional study showed a significant association between BUN and critical limb ischemia (CLI) in patients with peripheral arterial occlusive disease (PAOD). It also showed that BUN could be used to identify patients with atherosclerosis at a high risk of CLI (35). This study further confirmed that the BUN level is an independent risk factor for PAD. However, owing to sclerosis of the arteries, the ABI might be unreliable, especially in older patients and those with diabetes. Therefore, BUN is an easily identifiable, widely available, and inexpensive marker that can identify patients at high risk for vascular endpoints, such as PAD.

Circulating Hb plays a role in various physiological processes and pathophysiological pathways, including oxygen-carrying capacity, inflammatory processes, oxidative stress, and blood viscosity. The Hb level is also an important indicator for diagnosing anemia. The incidence of comorbid anemia was higher in patients with DM and PAD. A previous study noted that people with diabetes and anemia (compared with non-anemic diabetes) had more comorbidities (mostly hypertension and PAD), more frequent hospitalizations, and a higher risk of death (36). Furthermore, decreased Hb levels in patients with diabetes increase the risk of hospitalization and death, and anemia appears to be a risk factor for all-cause mortality (37). In addition, Desormais et al. showed that anemia is a strong and independent predictor of death and limb loss in patients hospitalized with PAD (38). The association was gradual, with an increased risk of amputation as the severity of anemia increased (38). Thus, there appear to be multiple adverse effects of anemia in patients with DM and PAD. The present study demonstrated that Hb is an independent risk factor for patients with T2DM and PAD and suggests that adequate attention should be given.

This study’s analysis shows that the nomogram is well-developed and has an accurate value for PAD prediction, as assessed by its discriminative ability. Therefore, the C-index of the training and validation sets were 0.765 and 0.765, and 0.716 and 0.716, respectively. The C-index of the nomogram in both the training and validation sets was >0.70, indicating that the model had good discrimination. A good fit was obtained by generating 1000 bootstrap samples to replace the original samples and repeating the entire modeling process to test and calibrate the calibration curve. Furthermore, the DCA curves of the training and validation sets showed that the nomogram model was more practical and accurate when the risk threshold was between 2.5% and 90% or between 3.5% and 88%. In conclusion, this study’s nomogram model shows a more accurate value for PAD prediction in patients with T2DM. Furthermore, its construction greatly improves clinical practicality, thereby significantly improving the precision of treatment selection in clinical practice.

To the best of our knowledge, this is the first study to identify the risk factors for PAD in patients with diabetes using a newly proposed nomogram with excellent diagnostic accuracy. However, this study had some limitations. First, all patient data were obtained from the same hospital. Although the nomogram model achieved good accuracy, there is still room for further prospective multicenter validation to confirm and improve the reliability of the nomogram and further increase its clinical utility. Second, this was a retrospective study. Hence, not all clinicopathological data were included in the study due to limited data availability, and additional potential risk factors need to be included. Third, only internal validation was performed in this nomogram prediction model. Hence, external validation is required to improve the predictive value of the nomogram model in the future.

In conclusion, after reviewing many studies and evaluating multiple variables, this study demonstrated that diabetes duration, BUN, and Hb levels are independent risk factors for PAD in patients with T2DM. In addition, this study established and validated a nomogram for PAD risk in patients with T2DM based on these findings. The model has high accuracy and good discriminative power for PAD prediction, which helps clinicians make targeted and active treatment plans for medical intervention promptly.

## Conclusions

In summary, this study found that the independent risk factors for PAD in patients with T2DM were the duration of diabetes and BUN and Hb levels. In addition, this study established a visual nomogram prediction model with good discriminatory power, calibration, and clinical utility to facilitate personalized clinical applications. It also facilitates the early prediction and identification of potentially high-risk patients with PAD in T2DM.

## Data availability statement

The raw data supporting the conclusions of this article will be made available by the authors, without undue reservation.

## Ethics statement

The study involving human participants was reviewed and approved by the Medical Ethics Committee of the Air Force Medical Center. Since this is a study strictly based on a clinical case registry, individual informed consent is not required.

## Author contributions

JL and SX conceived and designed the study. Data collection and data analysis were performed by JL JS,TS and LZ, JL, JS and SX drafted the manuscript. All authors contributed to the article and approved the submitted version.
